# The 3-D Structural Basis for the *Pgi* Genotypic Differences in the Performance of the Butterfly *Melitaea cinxia* at Different Temperatures

**DOI:** 10.1371/journal.pone.0160191

**Published:** 2016-07-27

**Authors:** Yuan Li, Stefan Andersson

**Affiliations:** Department of Biology, Lund University, Lund, Sweden; Oxford Brookes University, UNITED KINGDOM

## Abstract

Although genotype-by-environment interaction has long been used to unveil the genetic variation that affects Darwinian fitness, the mechanisms underlying the interaction usually remain unknown. Genetic variation at the dimeric glycolytic enzyme phosphoglucoisomerase (Pgi) has been observed to interact with temperature to explain the variation in the individual performance of the butterfly *Melitaea cinxia*. At relatively high temperature, individuals with *Pgi-non-f* genotypes generally surpass those with *Pgi-f* genotypes, while the opposite applies at relatively low temperature. In this study, we did protein structure predictions and BlastP homology searches with the aim to understand the structural basis for this temperature-dependent difference in the performance of *M*. *cinxia*. Our results show that, at amino acid (AA) site 372, one of the two sites that distinguish Pgi-f (the translated polypeptide of the *Pgi-f* allele) from Pgi-non-f (the translated polypeptide of the *Pgi-non-f* allele), the Pgi-non-f-related residue strengthens an electrostatic attraction between a pair of residues (Glu373-Lys472) that are from different monomers, compared to the Pgi-f-related residue. Further, BlastP searches of animal protein sequences reveal a dramatic excess of electrostatically attractive combinations of the residues at the Pgi AA sites equivalent to sites 373 and 472 in *M*. *cinxia*. This suggests that factors enhancing the inter-monomer interaction between these two sites, and therefore helping the tight association of two Pgi monomers, are favourable. Our homology-modelling results also show that, at the second AA site that distinguishes Pgi-f from Pgi-non-f in *M*. *cinxia*, the Pgi-non-f-related residue is more entropy-favourable (leading to higher structural stability) than the Pgi-f-related residue. To sum up, this study suggests a higher structural stability of the protein products of the *Pgi-non-f* genotypes than those of the *Pgi-f* genotypes, which may explain why individuals carrying *Pgi-non-f* genotypes outperform those carrying *Pgi-f* genotypes at stressful high temerature.

## Introduction

Although genotype by phenotype/environment interaction has long been used to unveil the genetic variation that affects Darwinian fitness [1–3], the mechanisms underlying the interaction usually remain unknown [4–6]. Studies about the 3-D structural (e.g. [7]), functional (e.g. [8–10]) and physiological (e.g. [11]) differences of the products of alternative alleles at the genes of interest are essential for understanding such mechanisms. Among these mechanistic studies, 3-D structural study of the translated protein products of genes is the most fundamental one.

The present study aims to understand the structural mechanism of the frequently observed interaction between temperature and the genotypes of the loci encoding the enzyme phosphoglucoisomerase (Pgi) (bPgi: protein; *Pgi*: gene) in the butterfly *Melitaea cinxia* (Linnaeus, 1758) (Lepidoptera: Nymphalidae). Pgi is a metabolic enzyme that catalyses the reversible isomerization between Glucose-6-phosphate (G6P) and Fructose-6-phosphate in glycolysis at a branching point of G6P that is also involved in several other metabolic pathways, e.g. gluconeogenesis [12]. Pgi is also known for its diverse moonlighting functions [13], such as acting as an autocrine motility factor [14] or as a neuroleukin [15]. Therefore, variation at Pgi may have many different physiological outcomes, however, in the present study, we will only focus on its primary glycolytic role in the energy metabolism, which provides fuel to the energetically demanding flight activity in butterflies [16]. Flight capacity is essential for *M*. *cinxia*’s foraging, escaping and reproduction, therefore difference in flight ability may have a significant effect on individual fitness [17, 18]. Pgi is not a regulating step of the glycolytic pathway, however, according to the “flux hypothesis”, Pgi protein variants that maximize pathway flux may be beneficial when there is an extreme demand of cellular energy, for example during peak flight metabolism or activities dependent on peak flight metabolism [6].

Genetic variation at Pgi in *M*. *cinxia* has been suggested to significantly influence individual performance (e.g. peak flight metabolic rate [18–20] and dispersal rate [19–21]), fitness components (e.g. fecundity [22]) and population dynamics [23, 24]. Interestingly, these influences are often found to be temperature-dependent. *M*. *cinxia* individuals with genotypes (hereafter referred to as the *Pgi-f* genotypes) involving one common *Pgi* allele, *Pgi-f* [20], shows a higher body temperature, a higher peak flight metabolic rate and a higher mobility than other genotypes (hereafter referred to as the *Pgi-non-f* genotypes) at low to moderate temperatures, while the opposite applies at high temperature (e.g. [11, 22, 25–27]). A similar *Pgi* genotype-temperature interaction is found for female oviposition and fecundity in *M*. *cinxia* [22, 28]. Female *M*. *cinxia* individuals typically fly to feed on nectar before they lay eggs, therefore stressful temperature that affects flight activity may also affect oviposition and fecundity [22, 28].

A trade-off between kinetic efficiency and thermal stability in the translated protein products of different *Pgi* genotypes has been observed in the biochemical studies of Pgi for *Colias* butterflies [8, 29, 30], for montane beetles [9] and for sea anemones [31]. A similar trade-off may explain the observed temperature dependence of the effect of *Pgi* genotypes on performance/fitness in *M*. *cinxia* [4]. More specifically, the protein products of the *Pgi-non-f* genotypes are expected to have higher thermal stability than those of the *Pgi-f* genotypes, while the opposite applies to kinetic efficiency. In partial agreement with this suggestion, an earlier experimental study of *M*. *cinxia* in Åland, SW Finland, shows that individuals with *Pgi-f*/*f* homozygotes are significantly less heat tolerant than those with other *Pgi* genotypes [11]. In the present study, we have homology-modelled the 3-D protein structures of Pgi protein variants in *M*. *cinxia*, and performed bioinformatic surveys of Pgi protein sequences in animals, with the aim to provide a structural explanation of the observed temperature dependence of the performance/fitness effect of *Pgi* genotypes in *M*. *cinxia*. Our findings may explain why *M*. *cinxia* individuals with *Pgi-non-f* genotypes outperform those with *Pgi-f* genotypes at stressful high temperature.

## Material and Methods

### The genetic architecture of the study system

The Åland populations of *M*. *cinxia* contain seven Pgi polypeptide variants (translated from *Pgi* alleles), among which Pgi-f is the second most common one [20, 32]. Characterization of the cDNA sequence of Pgi in *M*. *cinxia* shows that one *M*. *cinxia* Pgi polypeptide sequence has 557 amino acid (AA) sites and that Pgi-f and Pgi-non-f differ by two Pgi AA sites (111 and 372 [23]). AA site 111 segregates into residues Lys and Gln while site 372 segregates into His and Asp in the Åland populations of *M*. *cinxia*. Pgi-f has Gln111+His372, while Pgi-non-f has Lys111+Asp372 [23].

### 3-D protein structure homology modelling

Homology modeling methods provide very accurate 3-D protein structure prediction [33, 34] and can be used for drug design and site direct mutagenesis [35, 36]. In the present study, we used the SWISS-MODEL, one of the commonly used homology modeling methods [37, 38]. The homodimeric Pgi 3-D protein structure in *M*. *cinxia* was homology modelled using the automated mode in the SWISS-MODEL workshop. Two polypeptide sequences (GenBank accession nos. ACF57704 and ACF57696, S1 Table) that are the two most common polypeptide sequences for Pgi in *M*. *cinxia* [23] were used as input for the modelling. ACF57704 represents the Pgi-f polypeptide variant while ACF57696 represents the Pgi-non-f variant. A Pgi crystal structure from pig (Protein Data Bank (PDB) [39] code 1gzv.1 [40]) which has a polypeptide sequence identity of 73.86% and 74.04%, respectively, to ACF57704 and ACF57696 was used as the template for modelling *M*. *cinxia* Pgi 3-D protein structures. The overall quality of the homology-modelled protein structures was evaluated with the ProSA-web server [41] by comparing the z-scores [42, 43] estimated for the modelled *M*. *cinxia* Pgi structures to the z-scores of all the experimental 3-D protein structures deposited in PDB. The z-scores of the modelled structures were -11.14 (for ACF57696) and -10.88 (for ACF57704), which fall within the range of z-scores estimated for the x-ray determined protein structures of similar length in PDB (S1 Fig), indicating a satisfactory quality of modelled structures.

The two modelled 3-D protein structures of *M*. *cinxia* Pgi were visualized and compared with DeepView/Swiss-PdbViewer v. 4.1.0 [44, 45]. The solvent-accessible surfaces of the AA residues of interest were also calculated with DeepView/Swiss-PdbViewer.

### Bioinformatics

Our homology modelling above indicated that the AA variation at the Pgi AA site 372 in *M*. *cinxia* affects the interaction between a particular pair of inter-monomer AA sites, 373 and 472 (see Results for more information). To explore the possible structural importance of the interaction between *M*. *cinxia* Pgi AA sites 373 and 472, we performed NCBI BlastP (v. 2.2.32+) homology searches [46, 47] in the GenBank peptide sequence database, based on all non-redundant GenBank CDS translations+PDB+SwissProt+PIR+PRF but excluding environmental samples from WGS projects. One *M*. *cinxia* Pgi peptide (AA positions 363–482, centering on the peptide of AA positions 373–472) from one published Pgi polypeptide sequence (GenBank accession no. ACF57696) was used as the input for the BlastP searches of homologous sequences within both plants (NCBI taxonomy identification no. [taxid]: 3193) and animals (taxid: 33208; also known as Metazoa, Animalia or multicellular animals [48]). However, the acquired plant sequences from the searches were not considered further, because the influence of Pgi variation on plant fitness may be complicated, due to the fact that, in addition to having the same (cytosolic) Pgi as animals, land plants harbour one extra isozyme of bacterial origin in the plastids [49]. All the other searching parameters used their default setting (except the maximum number of target sequences, which was set to 20000). The acquired animal homologous sequences that had no alignment reported at either of the two Pgi AA sites (referred to as “Animal373” and “Animal472”) that are equivalent to *M*. *cinxia* Pgi AA sites 373 and 472 were excluded. Only one homologous sequence within each animal family was kept, except for families that each had more than one type of combinations of the AAs at Pgi AA sites Animal373 and Animal472, in which case one sequence of each type was kept. This step was added to avoid overrepresentation of AA combinations resulting from possible sharing of ancestral residues among closely related taxa. Homologous sequences that either had gap or ambiguous data at sites Animal373 and/or Animal472, were also removed. In total, 483 animal Pgi sequences (S2 Table) were remained for summarizing the frequency of each type of AA combinations at sites Animal373 and Animal472. The maximum E-value (http://blast.ncbi.nlm.nih.gov/Blast.cgi?CMD=Web&PAGE_TYPE=BlastDocs&DOC_TYPE=FAQ) for these 483 Pgi peptide sequences is 6×10^−19^, suggesting reliable identification of the Pgi AA sites Animal373 and Animal472.

In addition, we used all the experimental Pgi 3-D protein structures available at PDB (downloaded at 2016-01-09) to determine the minimum distance (in Å) between residues at Pgi AA sites corresponding to *M*. *cinxia* Pgi AA sites 373 and 472. One Pgi structure per species was measured using DeepView/Swiss-PdbViewer. 3-D protein structures of cupin-type Pgi was excluded from the analyses due to their completely different overall structure compared to the conventional Pgi (cf. [50]). 3-D protein structures with aligned gaps at the relevant sites were also excluded from the analyses. In total 44 Pgi structures from 16 species covering three kingdoms (Bacteria, Protista and Animalia) were examined.

### Statistical analyses

If the 20 naturally-occurring proteinogenic AAs [51] have an equal chance to occur at Pgi AA sites Animal373 and Animal472, then the two sites should have a 3.0%, 3.25% and 93.75% probability of possessing an electrostatically attractive, repulsive or “neutral” AA combinations, respectively. Electrostatically attractive AA combinations refer to cases where one of the two Pgi sites has an acidic residue (Asp or Glu, [51]) and the other has a basic one (Lys, His or Arg, [51]). Electrostatically repulsive AA combinations refer to cases where the AAs at the two sites are both acidic or both basic. “Neutral” AA combinations refer to the rest of the combinations of the 20 AAs.

We determined the frequencies of the three AA combination groups at sites Animal373 and Animal472 among the 483 Pgi peptide sequences acquired from BlastP searches, and then compared these frequencies with the expected frequencies using Chi-square test. In situations where one or more of the expected and/or observed frequencies was smaller than 5 (for which the Chi-square test is unreliable), Fisher’s exact test was used instead.

## Results

### Local structural comparison between the two AA residues at each of the Pgi AA sites 111 and 372 in *M*. *cinxia*

**Fig 1:**
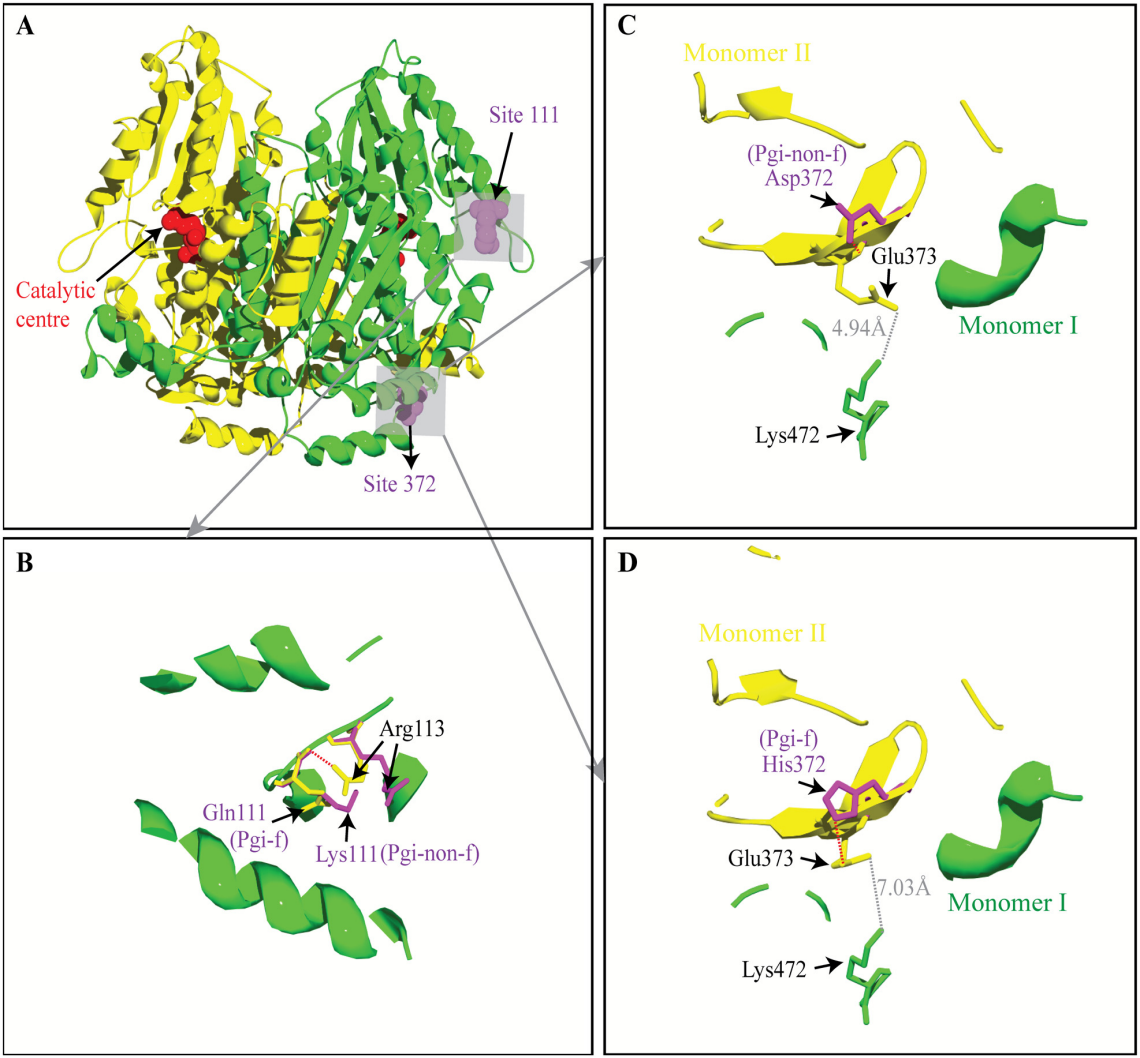
Homology-modelled *M*. *cinxia* Pgi structures showing the structural variation at AA sites 111 & 372. A) Pgi dimer with one monomer shown in yellow and the other shown in green. The two Pgi AA sites (111 and 372) of interest are shown in purple and a competitive inhibitor (5-phosphoarabinonate, in red) of the enzyme substrate indicates the locations of the catalytic centres. B) shows the AA residues that are within the 10Å distance of the AA site 111, which is located on a surface loop. At site 111, comparing to the neutral residue Gln111 (in yellow) of Pgi-f, the alternative basic residue Lys111 (in purple) of Pgi-non-f “pushes” the nearby basic residue Arg at site 113 (yellow when site 111 has Gln while purple when site 111 has Lys) away. Moreover, a hydrogen bond is formed between the Gln111 carbonyl oxygen atom and the side chain guanidino group of Arg113, while no hydrogen bond is found between Lys111 and Arg113. C) and D) show the AA residues within the 10Å distance of either His372 (in purple) of Pgi-f or Asp372 of Pgi-non-f (in purple). Site 372 is close to an inter-monomer interaction between Glu373 (in yellow) that is located in the same monomer (in yellow) as site 372 and residue Lys472 (in green) that is located in the other monomer (in green). Compared to the basic His372 of Pgi-f, the acidic Asp372 of Pgi-non-f “pushes” the acidic Glu373 closer to Lys472: the distances between GLu373 and Lys472 is 4.94 Å and 7.03 Å, respectively, when Asp372 and His372 occur. In addition, a hydrogen bond between the side chain carboxyl group of the Glu373 and the imidazole ring of His372 draws Glu373 away from the inter-monomer interface, while only the backbone amino group of Glu373 forms a hydrogen bond to the side chain carboxyl group of Asp372.

To investigate how the AA variation at the Pgi AA sites 111 and 372 that define the common polypeptide variant Pgi-f in *M*. *cinxia* affect the kinetic efficiency and/or thermal stability of the enzyme, we homology-modelled the homodimeric protein structures for the Pgi polypeptide variants Pgi-f (Gln111+His372) and Pgi-non-f (Lys111+Asp372). The modelled protein structures in the present study closely resembled the template 3-D structure as well as most other experimental Pgi 3-D structures (e.g. [52]). The functional unit of Pgi comprises two monomers (Fig 1A), and the catalytic centre, where the substrate binds and the chemical reaction takes place, are composed by residues from both monomers [53–55]. Therefore the proper association of the two monomers seems essential for the Pgi function.

Pgi AA site 111 segregates into two AA variants (Lys and Gln, which correspond, respectively, to Pgi-non-f and Pgi-f) in *M*. *cinxia* [23]. Our modelling results showed that site 111 is located on a loop at the surface (solvent-accessible surface 39–40% for the Lys111 of Pgi-non-f and 31–32% for the Gln111 of Pgi-f) of the Pgi structure. Compared to the neutral Gln111, the basic Lys111 “pushes” another nearby basic residue (Arg113) away (Fig 1B). In addition, a hydrogen bond found between the Gln111 carbonyl oxygen atom and the side chain guanidino group of Arg113 disappears when Lys occurs at site 111 (Fig 1B).

Pgi AA site 372 segregates into two AA variants (Asp and His) in *M*. *cinxia* [23]. The homology modelling results showed that Pgi AA site 372 resides on the inter-monomer boundary and is next to a likely electrostatically attractive interaction that is between an inter-monomer pair of Pgi residues: Glu373 is from the same monomer as site 372 while Lys472 is from the other monomer (Fig 1C and 1D). Compared to the weakly basic His372, the acidic Asp372 “pushes” Glu373 (also acidic) away, leading to a closer distance (4.94Å in the presence of Asp372 and 7.03 in the presence of His372) between Glu373 and Lys472 (Fig 1C and 1D) and therefore strengthening the electrostatic attraction between this inter-monomer pair. In addition, our result also showed that a hydrogen bond between the side chain carboxyl group of the Glu373 and the imidazole ring of His372 draws Glu373 away from the inter-monomer interface, while only the backbone amino group of Glu373 forms a hydrogen bond to the side chain carboxyl group of Asp372. The solvent accessible surfaces for His372 and its corresponding Glu373 are, respectively, 29–30% and 34–35% while the solvent accessible surfaces for Asp372 and its corresponding Glu373 are, respectively, 33–34% and 37–38%.

### AA variation at the Pgi AA sites Animal373 and Animal472

A majority of the 483 animal Pgi peptide sequences acquired from the BlastP searches came from two of the most species-rich phyla: Arthropoda (n = 291) and Chordata (n = 160) (https://simple.wikipedia.org/wiki/List_of_animal_phyla) [56]. Within Arthropoda, the acquired sequences were largely represented by the largest class in this phylum: insects [56, 57] (n = 245), while Chordata was dominated by three of the largest classes in this phylum [56]: Actinopterygii (ray-finned fishes, n = 36), Aves (birds, n = 49) and Mammalia (mammals, n = 59).

In total, 15 and 12 kinds of AAs were found, respectively, at Pgi AA sites Animal373 and Animal472. All the five electrically charged AAs (Arg, Lys, His, Glu and Asp) were found at site Animal373 but only three of these (Arg, Lys and Glu) were represented at site Animal472 (S3 Table). The proportions of charged AAs at both sites are, respectively, 60% (for Animal373) and 74% (for Animal472) (S2 Table). Seventy different combinations of the AAs at Pgi sites Animal373 and Animal472 were found among the 483 acquired animal Pgi sequences (S3 Table). The 70 AA combinations were grouped into three groups based on the electric charges of the two AA residues within each combination: 1) electrostatically attractive combinations (n = 5 types of AA combinations, S3 Table). 2) electrostatically “neutral” combinations (n = 63, S3 Table), and 3) electrostatically repulsive combinations (n = 2, S3 Table).

The total number of acquired Pgi sequences (across all analysed phyla) in the three AA combination groups was, respectively, 230 (for group 1), 248 (for group 2), and 5 (for group 3) (S3 Table), i.e. there was a substantial overrepresentation of electrostatically attractive combinations at sites Animal373 and Animal472. In the largest phylum, Arthropoda, 27% (n = 78) of the AA combinations were electrostatically attractive, among which Glu-Lys (Glu occurs at site Animal373 and Lys occurs at site Animal472; n = 47) and Arg-Glu (n = 19) predominated. In the largest class within Arthropoda (insects), 22% (n = 55) of the AA combinations were electrostatically attractive. Within the second largest phylum (Chordata) of the data, about 81% (n = 130) of the AA combinations were found to be electrostatically attractive, with a clear dominance of Arg-Glu (n = 128). The three main analysed classes within Chordata had, respectively, 33% (n = 12, for ray-finned fishes), 100% (n = 49, for birds) and 91% (n = 54, for mammals) electrostatically attractive AA combinations at sites Animal373 and Animal472.

**Fig 2:**
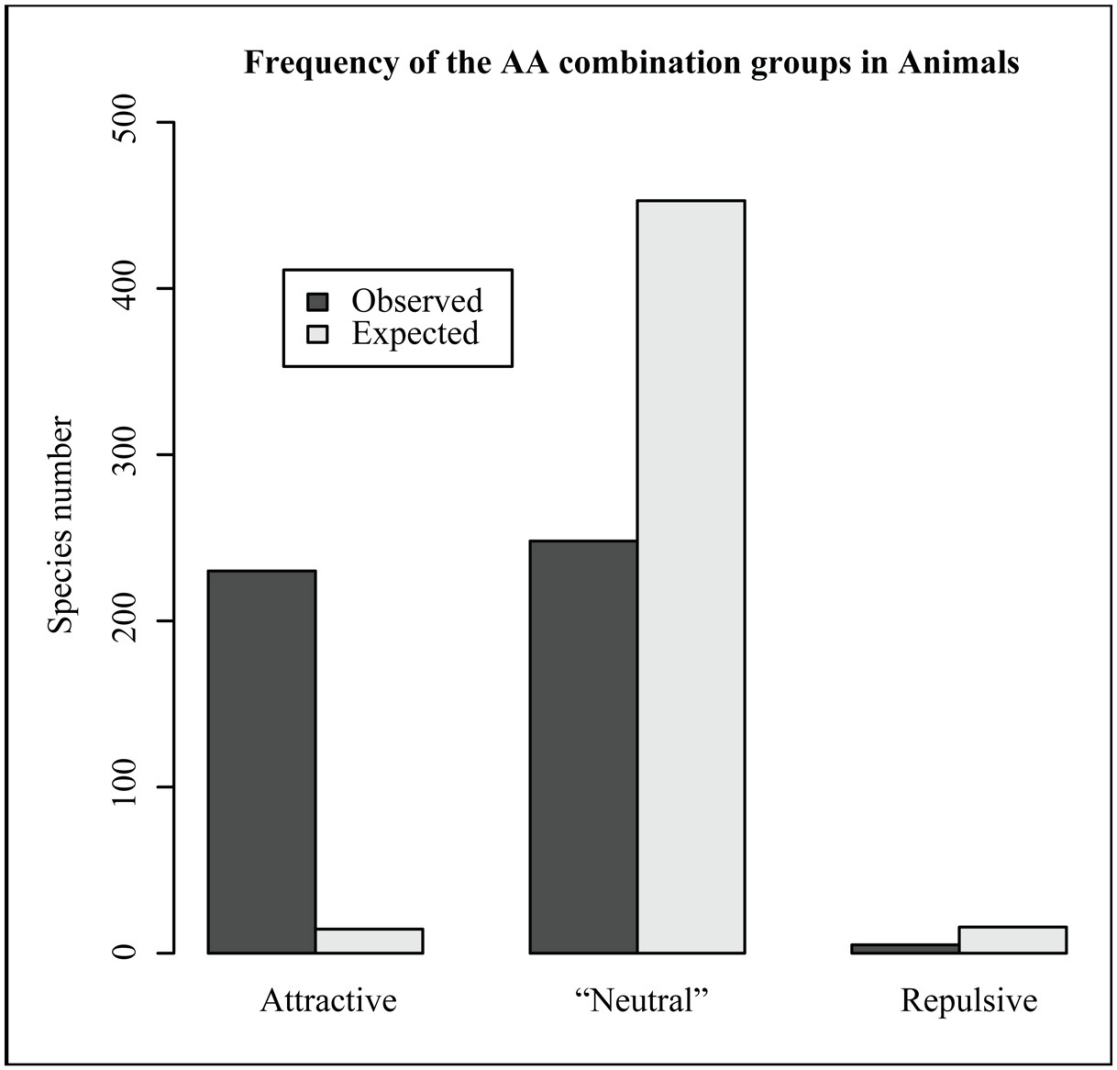
The overall frequencies of the AA combination groups at Pgi AA sites Animal373 and Animal472.

A Chi-square test comparing the observed and expected frequencies of different types of AA combinations gave a highly significant result for data pooled across phyla (X^2^ = 255.35, df = 2, *P* < 2.2×10^−16^, Table 1). This result is mostly due to an overall excess of electrostatically attractive combinations (Obs/Exp = 15.87) (Fig 2, Table 1). Fisher’s exact tests for comparing observed and expected frequencies of AA combination groups within the two largest phyla (Arthropoda and Chordata) and within the four main classes (insects, ray-finned fish, birds and mammals) showed a similar pattern (Table 1).

**Table 1:** Comparison of the observed and expected frequencies of the combination groups of the AAs at Pgi AA sites Animal373 and Animal472.

		AA combination groups at sites Animal373 and Animal472		
		I (Electrostatically attractive)	II (Electrostatically “neutral”)	III (Electrostatically repulsive)
Animals	Observed	230	248	5
	Expected	14.49	452.81	15.70
	X^2^ = 255.35, df = 2, *P* < 2.2×10^−16^***			
Arthropoda	Observed	78	210	3
	Expected	8.73	272.81	9.46
	Fisher’s exact test, *P* < 2.2×10^−16^***			
Chordata	Observed	130	29	1
	Expected	4.80	150.00	5.20
	Fisher’s exact test, *P* < 2.2×10^−16^***			
Insects	Observed	55	187	3
	Expected	7.35	229.69	7.96
	Fisher’s exact test, *P* = 1.48×10^−11^***			
Ray-finned	Observed	12	23	1
fishes	Expected	1.08	33.75	1.17
	Fisher’s exact test, *P* = 0.0013**			
Birds	Observed	49	0	0
	Expected	1.47	45.94	1.59
	Fisher’s exact test, *P* < 2.2×10^−16^***			
Mammals	Observed	54	5	0
	Expected	1.77	55.31	1.92
	Fisher’s exact test, *P* < 2.2×10^−16^***			

*** *P* < 0.001,

** 0.01 < *P* < 0.001

As a final step, we measured the distance between the Pgi AA sites equivalent to *M*. *cinxia* Pgi AA sites 373 and 472 for experimentally determined Pgi 3-D protein structures from Bacteria, Protista, and Animalia. The observed distances varied between 3-11Å, with the mammals having the shortest distance (3-4Å) (S4 Table).

## Discussion

*Pgi* is a gene that has been extensively investigated, especially in insects, for its substantial polymorphism and frequently observed genotype-phenotype/fitness associations [6, 58, 59]. Interestingly, these associations were sometimes found to be temperature-dependent (e.g. [22, 25]), suggesting a trade-off between the thermal stability and kinetic efficiency of the translated protein products of *Pgi* genotypes. Biochemical analyses of the Pgi enzyme activity in a number of species supported this hypothesis (e.g. [9, 29]). A number of studies have tried to understand the variation in the catalytic properties of the translated protein products of *Pgi* genotypes by examining the structural locations of the AA sites that distinguish between Pgi variants and/or that have been identified to be under selection (e.g. [60–62]). One study even attempted to investigate the 3-D structural differences between different AAs at the Pgi variant-distinctive AA sites, though giving no clear conclusion [7]. In the present study, we aim to explain the temperature dependence of the observed Pgi genotype-performance interactions in *M*. *cinxia* by examining the structural difference between the AA residues at two Pgi AA sites (111 and 372) that underlie Pgi protein variation in *M*. *cinxia*. Our results show that compared to the Pgi-f-defining residues at Pgi AA sites 111 and 372, the Pgi-non-f-defining residue at site 111 greatly decreases the hydrophobic area of Pgi surface at site 111, and the Pgi-non-f-defining residue at site 372 strengthens an electrostatically attractive inter-monomer interaction between Glu373 and Lys472. We also compared the *M*. *cinxia* Pgi peptide sequence with its animal homologous sequences in the GenBank protein databases. Our results indicate an excess of electrostatically attractive AA combinations at the AA sites (Animal373 and Animal472) of animal Pgi that are equivalent to *M*. *cinxia* Pgi AA sites 373 and 472.

### AA variation at *M*. *cinxia* Pgi sites 372 and 111

Pgi sites 372 and 111 in *M*. *cinxia* distinguish between the Pgi polypeptide variants Pgi-f and Pgi-non-f [23]. Two AA variants (His and Asp) at Pgi AA site 372 have been reported in *M*. *cinxia*. Our results show that site 372 is located on the interface between the two Pgi monomers and compared to variant His372 (corresponding to Pgi-f), the acidic Asp372 (corresponding to Pgi-non-f) “pushes” the neighboring acidic Glu373 (of the same monomer) closer to the basic Lys472 of the other monomer. This may strengthen the electrostatically attractive interaction between the inter-monomer residue pair Glu373 and Lys472, which very likely leads to an increase in the structural stability of Pgi in *M*. *cinxia*. Consistent with this suggestion, an earlier experimental study of *M*. *cinxia* from Åland showed that individuals with *Pgi-f/f* homozygotes were significantly less heat tolerant than those with other *Pgi* genotypes [11]. The stabilizing effect of Asp372 may help to explain why, at high temperature (thermal stress), the translated protein products of the *Pgi-non-f* genotypes surpass those of the *Pgi-f* genotypes as frequently reported (e.g. [22, 25]).

There are two sympatric catalytic centres within a Pgi dimer, each is composed by residues from both monomers [55]. *M*. *cinxia* Pgi AA sites 372 and 373 are located at a peptide (AA positions 362–391) that interconnects the two catalytic centres of a Pgi dimer [61] and penetrates through the interface between the two monomers. Part of this peptide has been frequently reported to have AA sites under positive/balancing selection [17, 61, 62], which, together with the special location of this peptide, may suggest that this peptide may be important not only for the structural stability but also for the kinetic efficiency of Pgi. It might be possible that an effect of the AA variation at site 372 on the kinetic efficiency of Pgi causes the protein products of the *Pgi-f* genotypes to have a higher kinetic efficiency than those of the *Pgi-non-f* genotypes, as suggested by the observations that *M*. *cinxia* individuals with *Pgi-f* genotypes have a better performance at relatively low temperature than those with *Pgi-non-f* genotypes (e.g. [22, 25]). In humans, charge-changing AA variation (at human Pgi AA sites 362 and 375) on the peptide that interconnects the two PGI catalytic centres has been shown to greatly affect the kinetic performance and/or thermal stability of the Pgi enzyme [63, 64].

Pgi AA site 111 resides on the surface of the Pgi structure. This study has shown that the change from a hydrophilic Lys111 (corresponding to Pgi-non-f) to hydrophobic Gln111 (corresponding to Pgi-f) greatly increases the hydrophobic area of the Pgi surface at site 111 and this entropy-unfavourable factor should decrease the structural stability of Pgi. The less hydrophobic area at the surface of Pgi-non-f-related protein structures around site 111 may also help explain why at high temperature (thermal stress), individuals with the *Pgi-non-f* genotypes surpass those with the *Pgi-f* genotypes. Moreover, site 111 is located on a loop (Fig 1A). Loops are a type of disordered structural regions, from which positively selected sites were frequently reported [62]. The disordered regions are believed to tolerate a high level of genetic variation and are therefore important for providing adaptive potentials [62, 65, 66].

Interestingly, though Pgi-f has been frequently reported to be beneficial to *M*. *cinxia* at low to intermediate temperatures, the Gln111/Gln111 and His372/His372 phenotypes (which together define the protein product of *Pgi-f/f* homozygote) are very rare (e.g. [11, 23]) in the Åland populations of *M*. *cinxia*. It might be that both Gln111 (compared to Lys111) and His372 (compared to Asp372) that define Pgi-f lead to higher kinetic efficiency of Pgi. There is a high level of linkage disequilibrium within the *Pgi* gene of *M*. *cinxia* [61], and the combined effects of both Gln111 and His372 for the *Pgi-f/f* homozygote might make the kinetic efficiency of Pgi too high to be good (for example, leading to overheating). However, the moderate increase in the kinetic efficiency as in the heterozygotes can still be beneficial, especially under highly energy-demanding activities. Consistent with this hypothesis, it has been shown that *M*. *cinxia* individuals of Åland populations with *Pgi-f* genotypes (mostly *Pgi-f/non-f* heterozygote) have higher body temperature after flight than those with *Pgi-non-f/non-f* homozygote [22].

### Inter-monomer interactions between Pgi AA sites Animal373 and Animal472

The functional unit of Pgi is a dimer comprising two properly combined monomers [55] and has an overall 3-D structure that is conserved among a wide range of organisms (e.g. [10, 53, 67, 68]). In our comparison of experimentally determined Pgi structures from different kingdoms, we found that Pgi AA site equivalent to *M*. *cinxia* site 373 of one Pgi monomer is located within the close vicinity of the Pgi AA site equivalent to *M*. *cinxia* site 472 of the other monomer (3Å-11Å). The four identified Pgi structures with electrostatically attractive AA combinations at Pgi AA sites equivalent to *M*. *cinxia* sites 373 and 472 represented mammals and had the shortest distances between the two sites (3-4Å) (S4 Table). The close distances between this pair of inter-monomer AA sites makes it possible for the residues at these sites to interact with high efficiency.

Our results show that electrostatically attractive combinations of the AAs at Pgi AA sites Animal373 and Animal472 are preferred not only when considering all the analysed animal Pgis as a whole, but also within each of the two main analysed animal phyla (Arthropoda and Chordata) and within each of the four main analysed animal classes (insects, ray-finned fishes, birds and mammals) (Fig 2 and Table 1). This result suggests independent multiple origins of electrostatically attractive inter-monomer interaction between residues at sites Animal373 and Animal472, probably caused by strong selection for increased structural stability of Pgi. The excess of electrostatically attractive AA combinations could be also because the residues at the two AA sites are conserved among different organisms and the electrostatically attractive AA combinations represent the ancestral status. However, this second possibility is unlikely. First the Pgi AA sites that are equivalent to sites Animal373 and Animal472 represent two of the most variable Pgi AA sites in a wide range of organisms (including animals), as suggested by the estimated Consurf normalized conservation scores [69] for these two sites (1.922 and 1.081) (Li Y, Hansson B, Ghatnekar L and Prentice HC, in preparation). Second, if the ancestral AAs at sites Animal373 and Animal472 are charged and tend to mutate to AAs of similar chemical characteristics [70], then, considering most of the five electrically charged AAs were found at both sites (S3 Table), there should be an excess of electrostatically repulsive AA combinations at these sites as well, which however is not the case (Fig 2).

Interestingly, among the two studied animal phyla, the evolutionarily more advanced Chordata has a much higher frequency of electrostatically attractive AA combinations at Pgi sites Animal373 and Animal472 (81%) than the less advanced Arthropoda (27%) (Table 1). Furthermore, within the three main Chordata classes analysed, the more evolutionarily advanced birds and mammals have much higher frequency of electrostatically attractive AA combinations at Pgi AA sites Animal373 and Animal472 (>90%) than the less advanced ray-finned fishes (ca. 50%). It is possible that the electrostatically attractive AA combinations at these two Pgi sites stabilize the metabolically essential Pgi enzyme, allowing advanced organisms to meet the demand of a complex body system and perhaps to colonize a broader variety of environments. The advantage of having stable enzymes (including Pgi) in the energy producing system may be particularly great in birds, given their highly energetically demanding flight activity.

### Conclusion and future perspectives

This study has investigated the possible structural mechanisms underlying the temperature dependence of the effects of the *Pgi* genotypes on the performance of the butterfly *M*. *cinxia* by homology modelling the Pgi 3-D protein structures of *M*. *cinxia*, and by surveying the AA components at a pair of Pgi AA sites within the animal kingdom. Our results show that, compared to the Pgi-f-defining residues within *M*. *cinxia*, the Pgi-non-f-defining residue Lys111 decreases the hydrophobic area of the Pgi structure surface at this site, and that the other Pgi-non-f-defining residue, Asp372, may strengthen an important, electrostatically attractive inter-monomer interaction in its vicinity. Our results suggest that both Pgi-non-f-defining residues may help strengthen the Pgi structural stability compared to the two Pgi-f-defining residues, which perhaps explain why individuals with *Pgi-non-f* genotypes perform better than those with *Pgi-f* genotypes at high temperatures. We advocate that similar 3-D structural studies may be performed to help better understand the previously observed genotype-environment interactions. 3-D structural studies of a molecule in a genotype-environment chain is just a fundamental step, mechanistic studies of subsequent steps such as the biochemical characterization of the molecule, physiological study of the organism may also need to be performed. Functional genomic studies have revealed that many other genes can be involved in the responses of an organism to environmental variation (e.g. [71]), so a comprehensive understanding of the genetic network underlying the adaptive response of an organism to its environment should be our final goal.

## Supporting Information

S1 Fig ProSA-web z-score plot showing the overall quality of the two modelled *M*. *cinxia* Pgi structures. A) and B) are, respectively, for the corresponding homodimeric 3-D protein structures of Pgi-non-f and Pgi-f. The z-scores [42, 43] of the two modelled protein structures in the present study are shown in black dots. In each panel, the light blue and dark blue dots show, respectively, the z-scores for all the 3-D protein structures in Protein Data Bank [39] that have been determined by X-ray analyses and nuclear magnetic resonance spectroscopy. The z-scores for the two modelled *M*. *cinxia* Pgi structures fall within the z-score ranges of the X-ray determined protein structures of similar numbers of residues in PDB.
(TIF)

S1 Table Amino acid (AA) polymorphism between the two most common *M*. *cinxia* Pgi polypeptide sequences that were used for homology modelling. The AA sites in bold are the two that can distinguish the polypeptide variants Pgi-f and Pgi-non-f.
(DOCX)

S2 Table The GeneBank accession numbers, taxon names of the 483 animal Pgi sequences that have been considered for summarizing the combinations of the amino acids (AAs) at Pgi AA sites Animal373 and Animal472, as well as the AA combination at these two sites for each sequence. (XLSX)

S3 Table Summary of the observed combinations of the amino acids (AA) at animal Pgi AA sites Animal373 and Animal472 within each combination group. (XLSX)

S4 Table The distances between the residues at Pgi amino acid (AA) sites equivalent to *M*. *cinxia* Pgi AA sites 373 and 472 within the experimentally determined Pgi 3-D protein structures from a wide range of organisms. (DOCX)
