# Supplementary figures and images for: The 3-D Structural Basis for the Pgi Genotypic Differences in the Performance of the Butterfly Melitaea cinxia at Different Temperatures
Source: PLoS One. 2016 Jul 27;11(7):e0160191. doi: 10.1371/journal.pone.0160191 (PMC4962976; doi:10.1371/journal.pone.0160191)

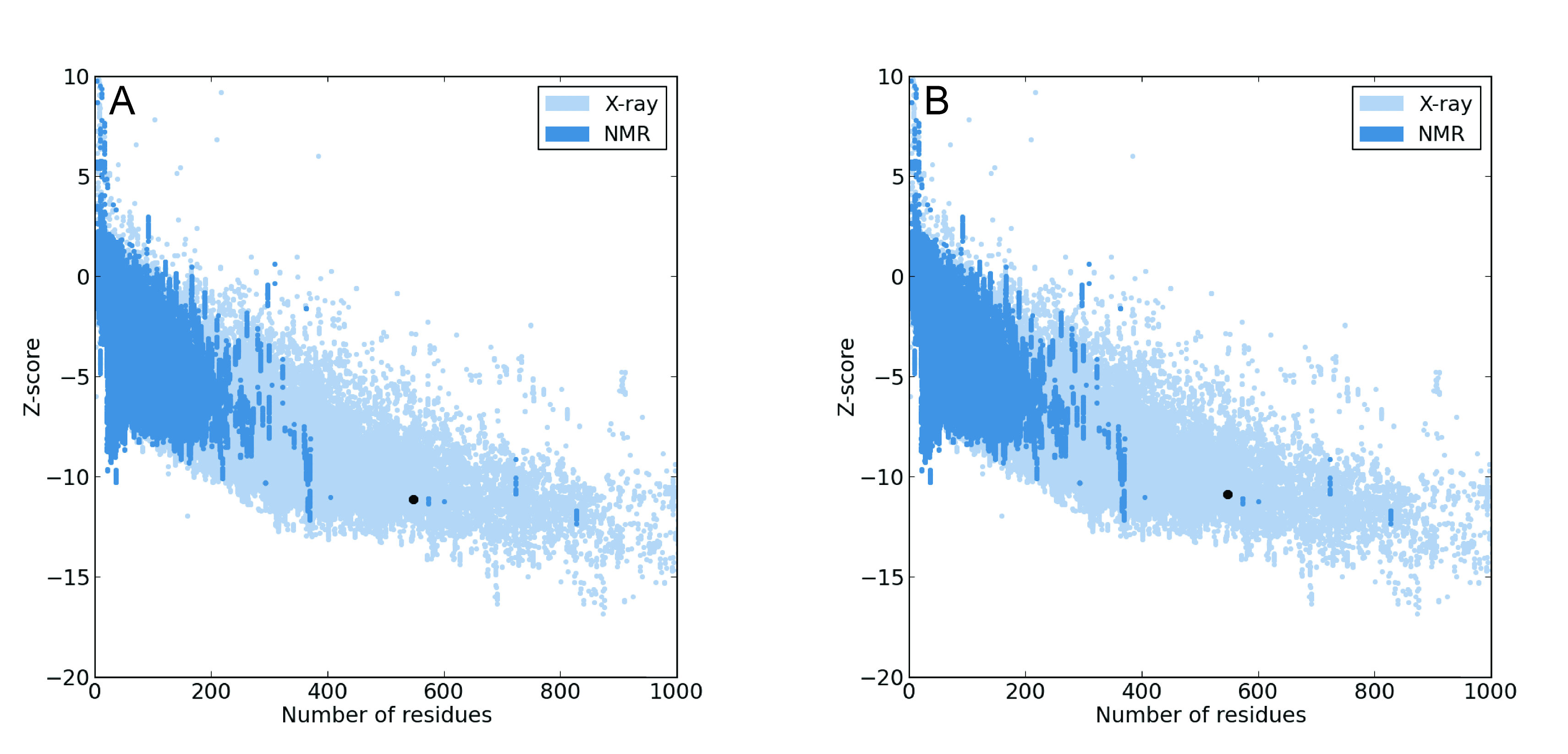

Supplement: S1 Fig — A) and B) are, respectively, for the corresponding homodimeric 3-D protein structures of Pgi-non-f and Pgi-f. The z-scores [42, 43] of the two modelled protein structures in the present study are shown in black dots. In each panel, the light blue and dark blue dots show, respectively, the z-scores for all the 3-D protein structures in Protein Data Bank [39] that have been determined by X-ray analyses and nuclear magnetic resonance spectroscopy. The z-scores for the two modelled M. cinxia Pgi structures fall within the z-score ranges of the X-ray determined protein structures of similar numbers of residues in PDB. (TIF) [file pone.0160191.s001.tif]
