# Supplementary material for: The 3-D Structural Basis for the Pgi Genotypic Differences in the Performance of the Butterfly Melitaea cinxia at Different Temperatures
Source: PLoS One. 2016 Jul 27;11(7):e0160191. doi: 10.1371/journal.pone.0160191 (PMC4962976; doi:10.1371/journal.pone.0160191)
Supplement: S1 Table — The AA sites in bold are the two that can distinguish the polypeptide variants Pgi-f and Pgi-non-f. (DOCX) [file pone.0160191.s002.docx]

**S1 Table. Amino acid (AA) polymorphism between the two most common *M*. *cinxia* Pgi polypeptide sequences that were used for homology modelling.** The AA sites in bold are the two that can distinguish the polypeptide variants Pgi-f and Pgi-non-f.

| GenBank accession no | Amino acid sites | | | | | | |
| --- | --- | --- | --- | --- | --- | --- | --- |
|  | 35 | 49 | 64 | **111** | 241 | 308 | **372** |
| ACF57696 (represents Pgi-non-f) | His | Met | Ile | **Lys** | Ala | Asn | **Asp** |
| ACF57704 (represents Pgi-f) | Gln | Thr | Val | **Gln** | Ser | Ser | **His** |
