# Supplementary material for: The 3-D Structural Basis for the Pgi Genotypic Differences in the Performance of the Butterfly Melitaea cinxia at Different Temperatures
Source: PLoS One. 2016 Jul 27;11(7):e0160191. doi: 10.1371/journal.pone.0160191 (PMC4962976; doi:10.1371/journal.pone.0160191)
Supplement: S4 Table — (DOCX) [file pone.0160191.s005.docx]

**S4 Table**. **The distances between the residues at Pgi amino acid (AA) sites equivalent to *M*. *cinxia* Pgi AA sites 373 and 472 within the experimentally determined Pgi 3-D protein structures from a wide range of organisms.**

| Kingdom | Species | Experimentally determined Pgi 3-D protein structure PDB codes§ | Residues at sites equivalent to *M*. *cinxia* Pgi AA sites | | Distance* |
| --- | --- | --- | --- | --- | --- |
|  |  |  | 373 | 472 |  |
| Animalia/Animals/Metazoa | Rabbit | 1dqr (1g98, 1hm5, 1hox, 1koj, 1n8t, 1xtb) | Arg369 | Glu468 | 3Å |
|  | Pig | 1gzd (1gzv) | Arg369 | Glu468 | 4Å |
|  | Human | 1jlh (1iat, 1iri, 1jiq, 1nuh) | Arg369 | Glu468 | 3Å |
|  | Mouse | 1u0e (1u0f, 1u0g, 2cvp, 2cxn, 2cxo, 2cxp, 2cxq, 2cxr, 2cxs, 2cxt, 2cxu) | Arg369 | Glu468 | 3Å |
|  | Alfalfa Butterfly | 4wmj | Thr373 | Lys472 | 7Å |
| Protista | *Leishmania mexicana* | 1t10 (1q50) | Ala422 | Thr519 | 8Å |
|  | *Trypanosoma brucei* | 2o2d (2o2c) | Thr423 | Ile521 | 7Å |
|  | *Plasmodium falciparum* | 3pr3 (3qki) | Pro392 | Asn490 | 7Å |
|  | *Trypanosoma cruzi* | 4qfh | Met424 | Thr522 | 8Å |
|  | *Toxoplasma gondii* | 3ujh | Thr375 | Pro469 | 8Å |
| Bacteria | *Thermotoga maritima* | 2q8n | Glu293 | Glu372 | 11Å |
|  | *Mycobacterium tuberculosis* | 2wu8 | Pro369 | Pro464 | 10Å |
|  | *Vibrio cholerae* | 3hjb | Pro368 | Glu465 | 6Å |
|  | *Francisella tularensis* | 3ljk (3m5p, 3q7i, 3q88) | Thr358 | Pro455 | 8Å |
|  | *Escherichia coli* | 3nbu | Val367 | Glu464 | 5Å |
|  | *Brucella melitensis* | 4em6 | Pro365 | Ser463 | 11Å |

§ In cases with more than one experimentally determined Pgi 3-D protein structures were found within one species, only the first one was examined for the distance between the residues at Pgi AA sites equivalent to *M*. *cinxia* Pgi sites 373 and 472, with the PDB codes of the rest of the structures shown in bracket.

* Distances are between the residues at Pgi AA sites equivalent to *M*. *cinxia* Pgi sites 373 and 472 within the examined Pgi protein structure.
